# Supplementary material for: Ring deconvolution microscopy: exploiting symmetry for efficient spatially varying aberration correction
Source: Nat Methods. 2025 Apr 29;22(6):1311–20. doi: 10.1038/s41592-025-02684-5 (PMC12165846; doi:10.1038/s41592-025-02684-5)
Supplement: Supplementary file 1 — Supplementary Information [file 41592_2025_2684_MOESM1_ESM.pdf]

nature methods

Supplementary information https://doi.org/10.1038/s41592-025-02684-5

Ring deconvolution microscopy: exploiting symmetry for efficient spatially varying aberration correction

In the format provided by the authors and unedited

1 Additional comparisons

This section describes the additional methods used in Extended Fig. 2 (main text). Two other commonly used deconvolution methods are Gaussian kernel deconvolution and modal decomposition. Gaussian kernel deconvolution involves fitting a two-dimensional Gaussian distribution to an experimental PSF and using the fitted kernel for deconvolution. While common, this method assumes spatial-invariance and cannot correct spatially-varying aberrations. On the other hand, modal decomposition is a spatially-varying deconvolution technique which models the spatially-varying image formation as a weighted sum of convolutions with space-invariant kernels. These kernels and their corresponding weights can be estimated from a uniformly spaced set of PSFs via singular value decomposition. We note that this type of calibration generally cannot be done single-shot and requires multiple acquisitions with a high precision motion stage. Since the multimode fiber experiment has such calibration information, we use it as a test bed for these additional techniques. It is also worth noting that that modal decomposition could be combined with our Seidel fitting procedure to further improve its accessibility; this exploration however is beyond the scope of this work and is left for future exploration.

2 Quantifying the performance of SDM

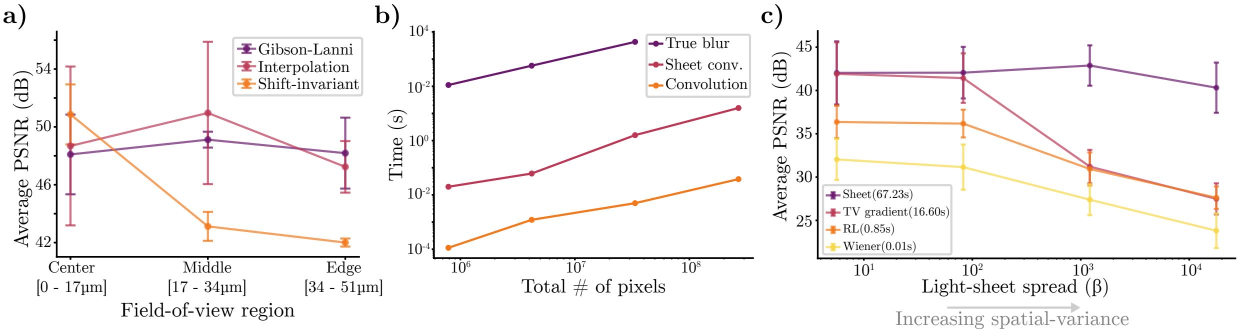

Figure 1: **Quantifying Sheet deconvolution microscopy. a)** Error evaluation of PSF fitting models. N=6 image stacks of randomly-scattered point sources are acquired with a light-sheet microscope and fit using either with the modified Gibson-Lanni model, by interpolating the measured PSFs, or by using the (denoised) center PSF and assuming shift-invariance. The PSNRs between the generated PSFs and the measured PSFs from the remaining calibration image stacks is computed for all three PSF models. These PSNRs are stratified by regions split along the light-sheet spread direction; center is where the light-sheet is the thinnest, while edge is where the light-sheet is the most spread. The resulting average PSNRs and their standard deviations in each region are plotted as a function of region. **b)** Forward model computation time for different data sizes. The runtimes of three different light-sheet forward models are plotted as a function of data size; true blur (manually superimposing the shift-varying PSFs at every point in the 3D volumes), sheet convolution, and standard 3D convolution. Times are acquired by processing randomly-generated 3D volumes and are averaged over 10 trials. **c)** Quantitative evaluation of sheet deconvolution. N=20 3D volumes of randomly sized and oriented ellipsoids are generated. These objects are blurred via true blur using PSFs generated from our modified Gibson-Lanni model, and are corrupted with Poisson noise (SNR 15) to simulate light-sheet measurements. The simulated measurements are deblurred with sheet deconvolution, iterative gradient-based deconvolution with total variation regularization, Richardson-Lucy deconvolution, and Wiener deconvolution. The process is repeated with increasing values of the β spread parameter, beginning with a system that is shift-invariant ranging to a system which is highly shift-variant due to rapid light-sheet spread. The average PSNR and its standard deviation over all 20 objects for each method is plotted as a function of the spread parameter.

The experimental evaluation of sheet deconvolution microscopy (SDM) highlights its resolution improvement and computational efficiency advantages. We further support these results by quantifying each part of

the SDM pipeline. Figure 1 details these quantitative experiments. First, quantify how well our light-sheet PSF fitting models, modified Gibson-Lanni and interpolation, fit experimental PSFs and compare them to the standard shift-invariant model. To that end, we fit these models to experimental PSFs acquired from a light-sheet microscope and compute the PSNR between generated PSFs and measured PSFs. We plot these PSNRs as a function of the PSF's location along the light-sheet spread, from the center, where the light-sheet is the thinnest, to the edges, where the light sheet is the most spread. Both of our models account for the shift-variance due to the light-sheet spread and consequently maintain accurate fitting across the entire FoV. In contrast, the standard shift-invariant model degrades away from the center, as expected. The interpolation model is higher variance than the modified Gibson-Lanni model; this is because the interpolation model is exact at the locations of the PSFs provided in the calibration image and worse in-between those PSFs, whereas the Gibson-Lanni fitting finds parameters that produce PSFs that fit well everywhere in the FoV, on average.

Next, we measure the runtime of the corresponding forward models, sheet convolution and 3D convolution, on different data sizes and find that, while sheet convolution is slower than 3D convolution, it is much faster than computing the true blur, i.e., manually superimposing the PSF at each point in the 3D volume. The scaling of runtime with data size of sheet deconvolution and 3D deconvolution can be inferred from these results but ultimately also depends on the sample and noise properties.

Finally, we quantify the accuracy of sheet deconvolution against standard deconvolution techniques, total variation (TV) iterative deconvolution, Richardson-Lucy deconvolution, and Wiener deconvolution. To do this we simulate phantom 3D samples (randomly sized and oriented ellipsoids) and simulate a light-sheet measurement using the true blur model with Poisson noise. We then deblur the simulated measurement with each method and compute the PSNR of the result with the ground truth sample. We repeat this process for systems with increasing light-sheet spread (increasing spatial variance). When the system is shift-invariant, sheet deconvolution performs as well as the best deconvolution technique, TV regularized iterative deconvolution. As the system becomes increasingly spatially-variant, the performance of sheet deconvolution remains relatively constant, while the performance of all the deconvolution methods degrades severely.

3 Simulation with Poisson noise

The quantitative experiments from the main text are rerun with Poisson noise instead of Gaussian noise (see Fig. 2). There are no notable differences in performances, with the same trends occurring as before: ring deconvolution is the best performing method, followed by DeepRD, the baseline U-Net, and finally standard deconvolution. Both deep learning models were updated with a small amount of training (100 iterations) on Poisson-noised images from the training set. Ring deconvolution and standard deconvolution were run with additional total variation regularization to account for the Poisson noise. We found that an SNR range of 15-30 most accurately reflected experimental data and thus ran all simulations with the worst-case SNR of 15.

4 Seidel Coefficients

Here, we give a brief background on Seidel coefficients. This is not a complete treatment and we encourage an interested reader to refer to the Voelz et al. matlab tutorial or the classic Born et al. book.

Consider a rotationally-symmetric—and consequently LRI—imaging system. It is common to consolidate the system aberrations into a single function and apply it at the exit pupil plane of the system. This complex-valued function, known as the generalized pupil function p is composed of a binary-valued amplitude distribution (set by the shape of the pupil) and a phase distribution w, which quantifies the deviation of the pupil wavefront from the ideal spherical shape necessary for diffraction-limited imaging. Since LRI systems are generally spatially-varying (albeit only radially), w becomes a function of the distance of the source from the optical axis r. Letting (s, t) be the pupil plane coordinates, we can write p as

$$p(s, t; r) = circ\left(\frac{r}{R}\right)e^{w(s,t;r)}.$$

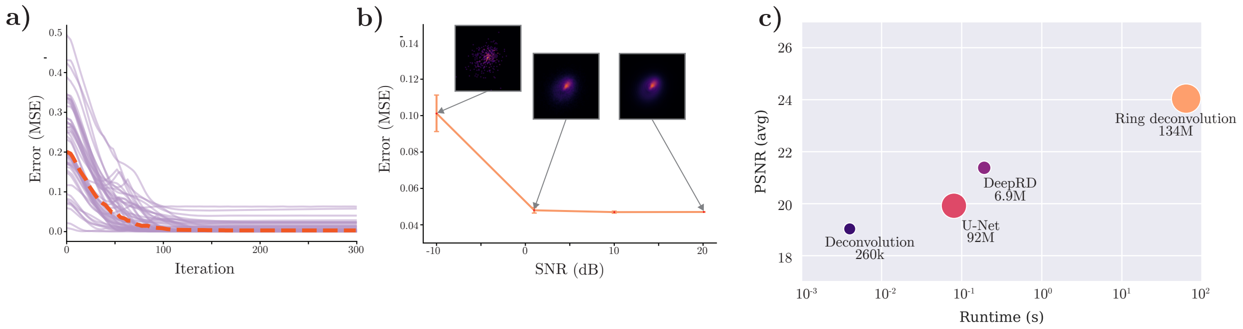

Figure 2: **Quantitative performance under Poisson noise.** **a)** A Poisson-noised image (SNR 15) of randomly placed PSFs is used to estimate the underlying Seidel coefficients, which can then be used to generate PSFs at any location. **e)** Seidel fitting error is plotted as a function of iteration in the optimization algorithm to demonstrate convergence. Each purple line is a different trial of n=50 trials with a randomly sampled set of underlying Seidel coefficients. The red dashed line is the per-iteration median. **b)** Mean squared error of the fitted Seidel coefficients plotted as a function of SNR of the calibration image. The average coefficient error is plotted along with the variance (error bar) over n=50 random trials. Some example calibration PSFs are shown. Even under severe noise, the Seidel fit is still accurate. **c)** Accuracy (PSNR) vs runtime of each method (averaged over n=28 true blurred images using unseen coefficients), with the number of model parameters (written below each circle) determining the size of the circle. Noise is Poisson distributed with SNR 15.

For rotationally-symmetric systems, as in our case, it is possible to expand w as an infinite power series. Usually only the 4th order terms of this series are used, which yields the following 2D polynomial of the pupil plane coordinates,

$$w_\omega(s,t;r) = \omega_s(s^2+t^2)^2 + \omega_c(s^2+t^2)sr + \omega_a s^2 r^2 + \omega_f(s^2+t^2)r^2 + \omega_d s r^3,$$

where $\omega = (\omega_s, \omega_c, \omega_a, \omega_f, \omega_d)$ are the five primary Seidel coefficients and w_ω only depends on the radial location due to the LRI assumption. Note that while the 5 primary Seidel coefficients are a subset of the infinitely many available coefficients, they represent the most common optical aberrations: spherical, coma, astigmatism, field curvature, and distortion. In particular, these aberrations are inherent to all spherically-shaped optics. Finally, the pupil function contains the same information as the PSFs, since they are related by a Fourier transform,

$$h(x,y;r) = \left|\mathcal{F}^{-1}\{p(-\lambda d f_x, -\lambda d f_y; r)\}\right|^2,$$

where λ is the wavelength of light, d is the distance from the pupil plane to the image plane, and f_x, f_y are the variables which the Fourier transform is taken over. Thus, knowledge of the 5 Seidel coefficients provides an approximation of the pupil function, which in turn, can accurately estimate PSFs for LRI systems, including the radial line of PSFs needed for computing ring convolution.

4.1 Higher-order coefficients

The Seidel series is an infinite power series expansion and thus has an infinite number of coefficients. While the aforementioned primary coefficients are dominant in practical optics—particularly for spherical lenses—it is worth considering the impact of higher-order coefficients, especially in the context of our Seidel fitting and ring convolution/deconvolution algorithms. To that end, we include a simulated experiment that tests our methods in the additional presence of 6th order Seidel aberration coefficients. In the experiment (shown in Fig. 3), a randomly-generated set of 13 (5 primary and 8 6th order) coefficients with unit norm are used to generate PSFs, which are then used to create a calibration image and blur a test image. The calibration image is noised and used to fit only the 5 primary coefficients. Finally, the fitted primary coefficients are used to deblur the blurry test image via ring deconvolution. Despite a noticeable difference in the higher-order PSFs, the fitting procedure almost always was able to converge to the correct primary coefficients, albeit with

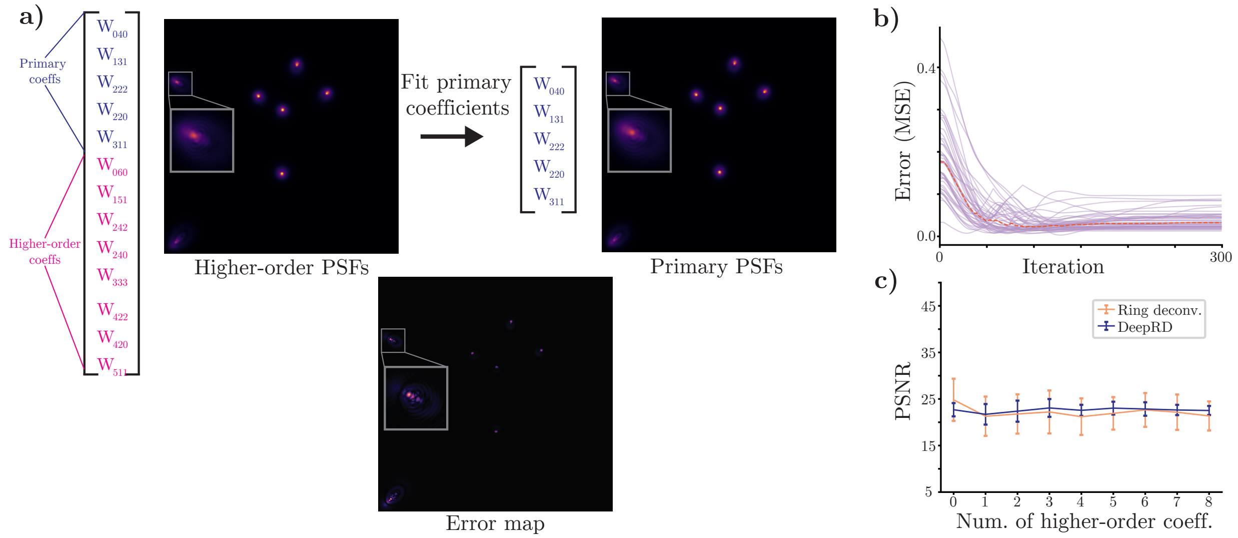

Figure 3: **Approximation of higher-order Seidel coefficients with primary Seidel coefficients. a)** An example trial: a random set of 13 Seidel coefficients are selected and used to generate a noisy calibration image. Next, only 5 primary Seidel coefficients are fit to the calibration image and are used to generate estimated PSFs. An error map between the high-order and fitted PSFs is shown at the bottom; some of the high-frequency features are missed by the lower-order approximation. **b)** Error in the fit between the estimated primary coefficients and true primary coefficients in the presence of higher-order coefficients. 50 trials are plotted with the red curve indicating the median convergence. The correct primary coefficients are estimated a majority of the time. We hypothesize that the small bump in the plot where the error slightly increases before convergence is an inflation of the primary coefficients to better explain the higher-order PSFs. **c)** Images blurred with higher-order PSFs are deblurred with RDM using lower order approximations of the PSFs and the PSNRs are recorded. The experiment is repeated with increasing degrees of higher-order blur, and thus increasing discrepancy from the lower-order approximations. The average PSNR and its standard deviation over 50 trials for ring deconvolution and DeepRD are plotted as a function of the number of higher-order coefficients used in the blur, from 0 (baseline case) to 8 (all 13 coefficients). While the average PSNRs for ring deconvolution seem to drop slightly from the baseline, all orders for both methods have PSNRs within a standard deviation of the baseline error, suggesting the presence of higher-order coefficients are not enough to drastically affect RDM. For all experiments, noise was Poisson with SNR 30 and the coefficients were always normalized to unit norm.

a slightly inflated values—likely accounting for the effects of the higher-order terms. The average deblurring error for ring deconvolution drops slightly compared to the baseline case (0 high-order coefficients), but is within a standard deviation of the baseline error. DeepRD's error also remains virtually unchanged and appears even more robust to the addition of high-order coefficients. Thus even in the presence of higher-order aberrations, RDM calibrated with only the primary Seidel coefficients produces accurate deblurring. As a final note, though the primary coefficients are largely sufficient, we do additionally include the option to fit the higher-order terms in our codebase.

5 Interpretability of DeepRD

One desirable property of DeepRD is its interpretability. Because the DeepRD latent space lives in the space of Seidel coefficients, we can easily see *why* our image reconstruction looks the way it does. Moreover, if our reconstruction is deficient, we can manually adjust the latent space, thereby changing the Seidel coefficients and improving the reconstruction. In the literature on encoder-decoder frameworks, this property is called disentanglement, that is, each element in the latent space has its own distinct and interpretable meaning. For example, if we only increase the coefficient value for radial distortion, then we expect the network to

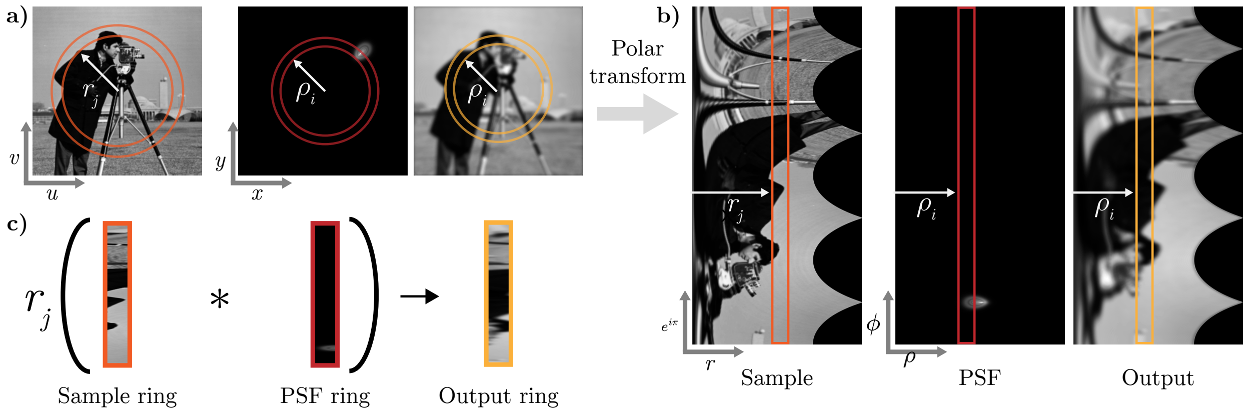

Figure 4: **Ring convolution.** A single step of the ring convolution algorithm. Here we are solving for a single concentric ring of the output denoted by its distance ρ_i from the center. **a)** Left to right: sharp 'sample', the system PSF at radius r_j, and blurry output of ring convolution.**b)** Corresponding polar resamplings of the images in a), this allows us to extract rings. **c)** The output ring at ρ_i is the sum of 1D convolutions; the ρ_i ring of the PSF at position r_j is convolved with the r_j ring of the sample for every radius r_j.

further unwarp the predicted image correspondingly.

This disentanglement property emerges in practice. We observe this by deblurring images of insect cornea taken by the Miniscope using a sweep over each aberration coefficient independently. For each coefficient, we set all other coefficients to 0 and sweep the chosen coefficient from 0 to 3 waves. What we observe is that the network only corrects for the chosen aberration in increasing amounts, while leaving the other characteristics of the image alone. For example, when we sweep distortion, the image remains blurry, but radially warps in the opposite direction compared to if it were distorted. Results for each aberration coefficient are shown in video form here. Such results confirm our hypothesis that DeepRD is indeed learning to perform ring deconvolution given a system's Seidel coefficients, and does not rely on knowledge of the specific image distribution. This further aids our confidence in its ability to generalize.

6 A Fourier analysis of LRI systems

Perhaps the main reason to make the linear space-invariant assumption is access to its Fourier space interpretation. Through an application of the Convolution Theorem to the space-invariant forward model, we see that an LSI system's output spectrum is a product of the input's spectrum with the spectrum of the impulse response, i.e., the transfer function. Thus any LSI imaging system can be thought of as a *filter* which individually scales each frequency component of the input object's intensity distribution. In the context of imaging, the transfer function—called the Optical Transfer Function (OTF)—describes how the imaging system scales each spatial frequency in the sample. The OTF gives rise to valuable intuitions; for example, since all practical imaging systems have bandlimited transfer functions, the output image is a lowpass-filtered version of the sample whose maximum-achievable resolution is directly proportional to the bandlimit. Moreover system aberrations can be analyzed by comparing the OTF in the presence of aberrations with the ideal OTF.

In this section we will formally develop an analogous Fourier interpretation of ring convolution and explain how its features can similarly be used to characterize the performance of an imaging system. While the LRI interpretation is more complicated than its LSI counterpart, it still provides a rich and realistic view of how LRI imaging systems transmit frequencies. As an example of its utility, we will see that the LRI model allows for a more general, radially-dependent notion of system resolution. For a review of the ring convolution operation please refer to Fig. 4.

To begin, we will define a slightly different notion of spatial frequency, called rotational frequency and denoted Θ, which is the quantity that gets filtered by an LRI system.

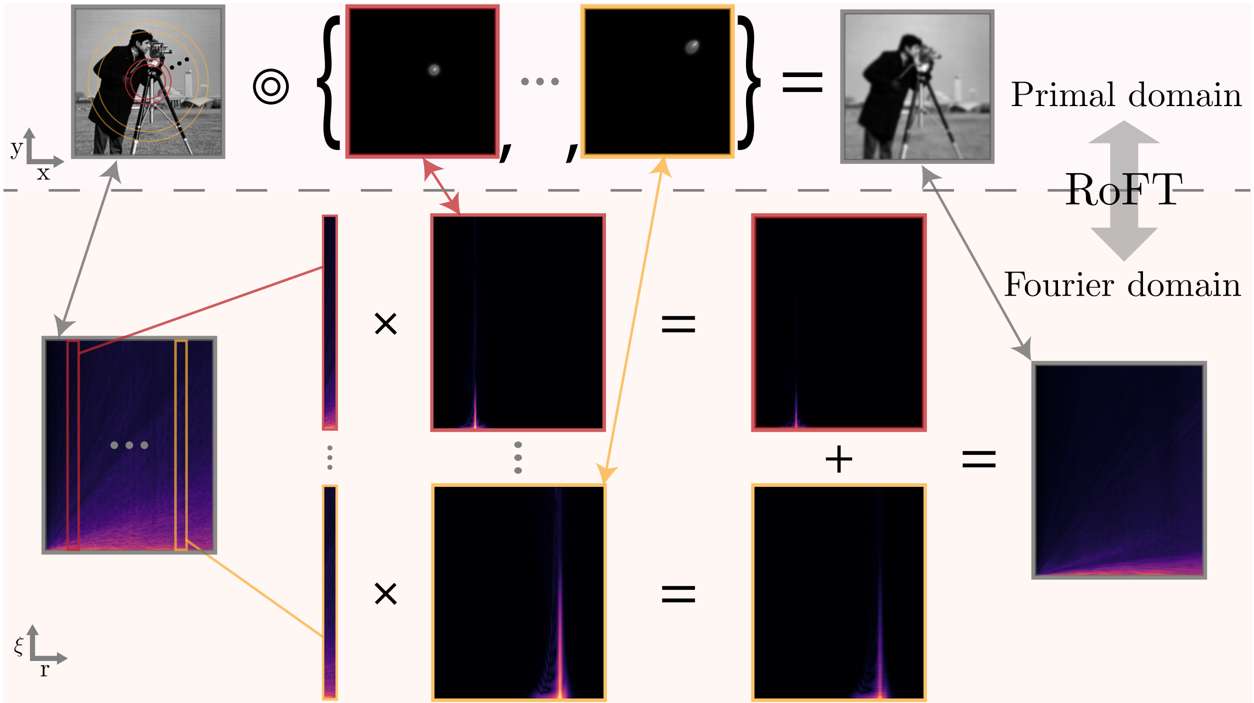

Figure 5: **LRI filtering.** The top, pink box shows LRI filtering in the spatial domain: an object (left) is LRI filtered by a radial set of PSFs (middle) to give a blurry image (right). The bottom, orange box shows the Fourier domain equivalent. Double arrows indicate Rotational Fourier Transform (RoFT) pairs. Strips at each r of the object RoFT (left) are individually multiplied by the RoFTs of the corresponding PSF at r (middle left) producing filtered contributions (middle right). Finally each of these contributions for every r is summed to form the RoFT of the blurry image (right).

Definition 1 (Rotational Fourier Transform (RoFT)). *Let $f : \mathbb{R}^2 \to \mathbb{C}$ represent a (potentially complex-valued) image, and let $\tilde{f}$ be it's polar counterpart. The RoFT of f is given by*

$$\tilde{F}(r, \xi) = \int \tilde{f}(r, \theta) e^{i2\pi\theta\xi} d\theta.$$

Intuitively, one can think of the values of rotational frequencies as quantifying how quickly the image can change as one travels in a ring of radius r around the center. However, this notion of oscillation speed depends on the radius—think about the spokes on the wheel of a bicycle, even though the spokes are evenly spaced for any given radius, they become further spaced for larger radii. However, under polar sampling they are scaled to have the same frequency. This scaling can be seen in Fig. 5 as the RoFT of each quantity tend to have higher frequencies at larger radii.

Now, writing the Ring Convolution Theorem in terms of the RoFTs of each quantity (denoted with capital letter and tilde), we see that

$$\tilde{F}(\rho, \xi) = \int r\tilde{G}(r, \xi)\tilde{H}(\rho, \xi; r) dr.$$

Like its LSI counterpart, the above model can be thought of a filtering operation, but now a more complex one; the object's values r away from the center are *filtered* and *mixed* by the rth PSF. To more fully understand this interpretation, consider the object's values at some radius r; they form a ring which will be filtered by the rth PSF. Specifically, the spectrum of this ring, or equivalently its RoFT at r, is point-wise multiplied by each ring in the RoFT of the rth PSF, which yields a set of filtered rings indexed by ρ. The ρth filtered ring represents the contribution of the object ring at r to the image ring at ρ (see Fig. 5).

It follows that the shape of the rth PSF determines both how much the object's rotational frequencies are filtered through the system and which parts of the object are mixed together to form the image. By

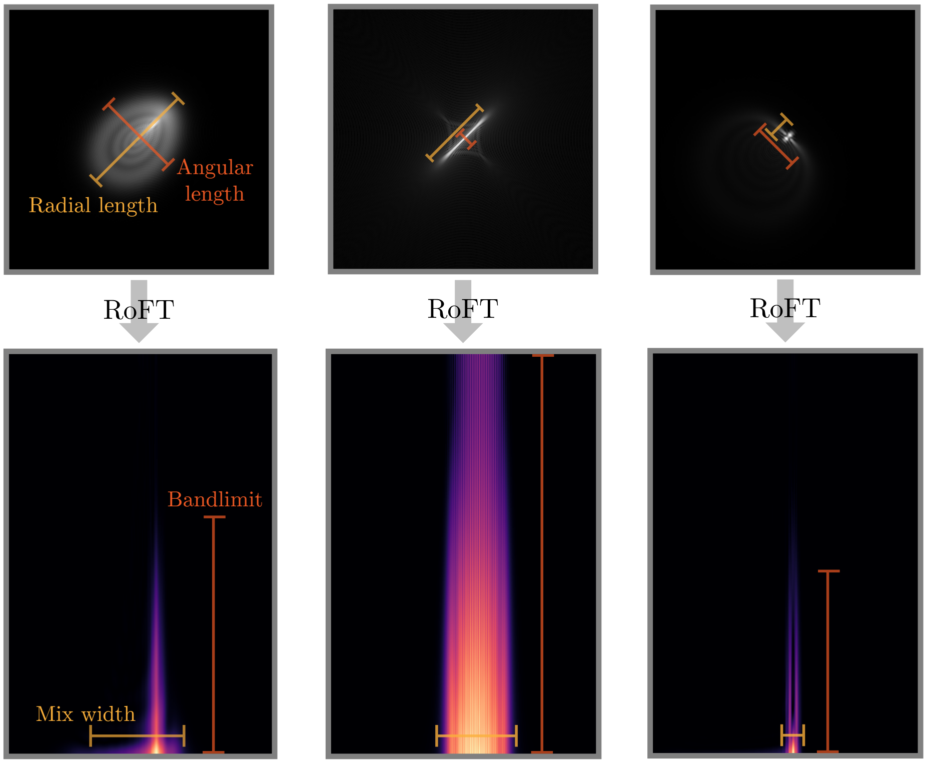

Figure 6: **LRI PSF interpretation.** Evaluating an LRI optical system amounts to considering the length of each PSF in the angular and radial directions. These lengths are inversely proportional to the bandwidth and mix width of the corresponding RoFT, respectively (see leftmost column). In the middle column we see an elogated PSF in the radial direction (i.e., astigmatism) which has a large bandwidth, but incurs more off-radius mixing due to a large mix width. The rightmost column shows an elongated PSF in the angular direction which has a small mix width, but has a relatively small bandwidth.

looking at the shape of the rth PSF we can determine the exact nature of this filtering and mixing. As shown in Fig. 6 the angular length of the PSF determines the height of the PSF's RoFT at ρ, which tells us which of the object rotational frequencies at r will make it to the image at ρ—this is analogous to the OTF bandwidth in the LSI case. Meanwhile, the radial extent of the PSF (i.e., how many concentric rings it covers) controls the extent of the image rings (i.e, which ρ values) are effected by the object at r. This manifests as the width of the PSF's RoFT which we call the *mix width*.

Now our notion of resolution, assuming the ability to perfectly de-mix, is radially dependent and, at radius r, is proportional to the angular arc length at $\rho = r$ of the rth PSF. The LRI filtering interpretation offers a more realistic understanding of how an imaging system filters an object and opens the door for a host of new imaging techniques which optimize for key features, such as resolution, under the LRI model. An analogous analysis for systems with other forms of symmetry— like the linear symmetry present in light-sheet microscopy—will lead to similar conclusions.
