# Supplementary material for: Ring deconvolution microscopy: exploiting symmetry for efficient spatially varying aberration correction
Source: Nat Methods. 2025 Apr 29;22(6):1311–20. doi: 10.1038/s41592-025-02684-5 (PMC12165846; doi:10.1038/s41592-025-02684-5)
Supplement: Supplementary file 2 — Reporting Summary [file 41592_2025_2684_MOESM2_ESM.pdf]

nature portfolio

Corresponding author(s): Amit Kohli

Last updated by author(s): Feb 21, 2025

Reporting Summary

Nature Portfolio wishes to improve the reproducibility of the work that we publish. This form provides structure for consistency and transparency in reporting. For further information on Nature Portfolio policies, see our Editorial Policies and the Editorial Policy Checklist.

Statistics

For all statistical analyses, confirm that the following items are present in the figure legend, table legend, main text, or Methods section.

n/a	Confirmed	
☐	☒	The exact sample size (*n*) for each experimental group/condition, given as a discrete number and unit of measurement
☐	☒	A statement on whether measurements were taken from distinct samples or whether the same sample was measured repeatedly
☒	☐	The statistical test(s) used AND whether they are one- or two-sided<br>*Only common tests should be described solely by name; describe more complex techniques in the Methods section.*
☒	☐	A description of all covariates tested
☒	☐	A description of any assumptions or corrections, such as tests of normality and adjustment for multiple comparisons
☐	☒	A full description of the statistical parameters including central tendency (e.g. means) or other basic estimates (e.g. regression coefficient) AND variation (e.g. standard deviation) or associated estimates of uncertainty (e.g. confidence intervals)
☒	☐	For null hypothesis testing, the test statistic (e.g. *F*, *t*, *r*) with confidence intervals, effect sizes, degrees of freedom and *P* value noted<br>*Give P values as exact values whenever suitable.*
☒	☐	For Bayesian analysis, information on the choice of priors and Markov chain Monte Carlo settings
☒	☐	For hierarchical and complex designs, identification of the appropriate level for tests and full reporting of outcomes
☒	☐	Estimates of effect sizes (e.g. Cohen's *d*, Pearson's *r*), indicating how they were calculated

Our web collection on statistics for biologists contains articles on many of the points above.

Software and code

Policy information about availability of computer code

Data collection	Data was collecting using Micromanager v1.4 accompanied by Pycromanager, Nikon NIS Elements Software (version 6.9.0), and LabView 2016.
Data analysis	All code can be found in our github repository: https://github.com/apsk14/rdmpy<br>Python 3.8.1 was used with the following packages:<br>* numpy 1.20.2<br>* pytorch 2.4.1<br>* scipy 1.6.2<br>* scikit-image 0.17.2<br>* pillow 8.2.0<br>* matplotlib 3.2.2<br>* tqdm 4.65.0<br>* kornia 0.5.3<br>* jupyter 1.0.0<br><br>ImageJ 1.53a was also used for psuedocoloring for display

For manuscripts utilizing custom algorithms or software that are central to the research but not yet described in published literature, software must be made available to editors and reviewers. We strongly encourage code deposition in a community repository (e.g. GitHub). See the Nature Portfolio guidelines for submitting code & software for further information.

Data

Policy information about availability of data

All manuscripts must include a data availability statement. This statement should provide the following information, where applicable:

- Accession codes, unique identifiers, or web links for publicly available datasets
- A description of any restrictions on data availability
- For clinical datasets or third party data, please ensure that the statement adheres to our policy

The data used in all of the imaging experiments (Miniscope, multicolor fluorescence, multimode fiber, and light-sheet) is publicly available on Box (https://berkeley.box.com/s/zmsjjgmquwq2roh4d9qthcnv3rhwuidn). Additional experimental data from the multimode fiber system can be requested from Turcotte et al. (https://opg.optica.org/boe/fulltext.cfm?uri=boe-11-8-4759&id=433935). The datasets used to train and fine-tune DeepRD, and to evaluate the quantitative performance of the methods are also hosted on Box (https://berkeley.box.com/s/vv3g6avhrr9agijmlj3b1153oo7x9gao). These datasets were sourced from the CARE dataset Weigert et al. (https://publications.mpi-cbg.de/publications-sites/7207/) and the Div2k dataset Agustsson et al. (https://data.vision.ee.ethz.ch/cvl/DIV2K/). The high resolution pretraining dataset, due to its large memory useage, will be made available upon request.

Human research participants

Policy information about studies involving human research participants and Sex and Gender in Research.

Reporting on sex and gender	Not applicable
Population characteristics	Not applicable
Recruitment	Not applicable
Ethics oversight	Not applicable

Note that full information on the approval of the study protocol must also be provided in the manuscript.

Field-specific reporting

Please select the one below that is the best fit for your research. If you are not sure, read the appropriate sections before making your selection.

☒ Life sciences ☐ Behavioural & social sciences ☐ Ecological, evolutionary & environmental sciences

For a reference copy of the document with all sections, see nature.com/documents/nr-reporting-summary-flat.pdf

Life sciences study design

All studies must disclose on these points even when the disclosure is negative.

Sample size	All samples in this study were individually imaged and processed independently. The methods presented are not statistical in nature and no statistics over the samples were relevant or reported. Sample sizes were chosen manually by the authors to ensure a sufficient, non-redundant display of the method.
Data exclusions	No data was excluded from the manuscript
Replication	The data acquisition and processing protocol was done by different authors in different locations at different times. In each case, the method proved to be successful with consistent performance across the different acquisition environments and imaging modalities. Code is provided to reproduce our main results.
Randomization	Samples/organisms were selected in a psuedorandom fashion by iterating over a large batch of the sample/organism and selecting individual images that maximized biologically relevant content. Since the method did not report any statistical attributes of the data, there was no specific criteria for sample/organism selection. All model data was randomly split into training, validation, and test sets.
Blinding	Blinding was only relevant in the context of the DeepRD model, which was only tested on unseen experimental data or an unseen synthetic test set.

Reporting for specific materials, systems and methods

We require information from authors about some types of materials, experimental systems and methods used in many studies. Here, indicate whether each material, system or method listed is relevant to your study. If you are not sure if a list item applies to your research, read the appropriate section before selecting a response.

Materials & experimental systems

n/a	Involved in the study
☒	☐ Antibodies
☐	☒ Eukaryotic cell lines
☒	☐ Palaeontology and archaeology
☐	☒ Animals and other organisms
☒	☐ Clinical data
☒	☐ Dual use research of concern

Methods

n/a	Involved in the study
☒	☐ ChIP-seq
☒	☐ Flow cytometry
☒	☐ MRI-based neuroimaging

Eukaryotic cell lines

Policy information about cell lines and Sex and Gender in Research

Cell line source(s)	The cells imaged were from the bovine pulmonary artery endothelial (BPAE) cell line (CVCL_4130) and were sourced from ThermoFisher (https://www.thermofisher.com/order/catalog/product/F36924). The other cells used were from the SU.86.86 cell line sourced from ATCC: CRL-1837 (https://www.atcc.org/products/crl-1837).
Authentication	Cells were authenticated by ThermoFisher. The authors did not authenticate them before imaging.
Mycoplasma contamination	To the author's knowledge, the cells were not tested for Mycoplasma contamination.
Commonly misidentified lines (See ICLAC register)	No commonly misidentified lines were used.

Animals and other research organisms

Policy information about studies involving animals; ARRIVE guidelines recommended for reporting animal research, and Sex and Gender in Research

Laboratory animals	Mixed-staged adults of the eutardigrade species Hypsibius exemplaris Z151 (reclassified from Hypsibius dujardini in 2017), purchased from Sciento (Manchester, United Kingdom). Ages ranged from 3 to 6 weeks.
Wild animals	The study did not involve wild animals.
Reporting on sex	Sex-based analysis is not relevant for this study. The technique presented is about deblurring images taken with aberrated systems and is agnostic to the sample being imaged.
Field-collected samples	The study did not involve samples collected from the field.
Ethics oversight	No ethical approval or guidance is required for tardigrades.

Note that full information on the approval of the study protocol must also be provided in the manuscript.
